# Shenling Baizhu powder alleviates non-alcoholic steatohepatitis by reducing PPARα-mediated NLRP3 inflammasome activation

**DOI:** 10.3389/fimmu.2026.1797561

**Published:** 2026-08-20

**Authors:** Siyuan Huang, Yuanjun Deng, Dajin Pi, Fangyu Zhou, Jinyue Pan, Qingliang Song, Lianghao Liu, Yuanyou Li, Qinhe Yang, Maoxing Pan, Yupei Zhang

**Affiliations:** 1 School of Traditional Chinese Medicine, Jinan University, Guangzhou, Guangdong, China; 2 Department of Hepatology, Guangdong Provincial Hospital of Chinese Medicine, Zhuhai, Guangdong, China; 3 State Key Laboratory of Traditional Chinese Medicine Syndrome, The Second Affiliated Hospital of Guangzhou University of Chinese Medicine, Guangzhou, Guangdong, China; 4 Postdoctoral Research Center, Guangdong Provincial Hospital of Chinese Medicine, Guangzhou, Guangdong, China; 5 The Second Clinical College of Guangzhou University of Chinese Medicine, Guangzhou, Guangdong, China; 6 School of Pharmaceutical Sciences, Guangzhou University of Chinese Medicine, Guangzhou, Guangdong, China

**Keywords:** NLRP3, nonalcoholic steatohepatitis, PPARα, Shenling Baizhu powder, TCM

## Abstract

**Introduction:**

Nonalcoholic steatohepatitis (NASH) is the progressive stage of nonalcoholic fatty liver disease characterized by varying degrees of inflammation, hepatic steatosis, liver cell damage and fibrosis, which can seriously harm people’s health. Shenling Baizhu (SL) powder exhibits a strong capacity to modulate cellular mechanisms, thereby demonstrating exceptional potential in addressing inflammatory and oxidative stress-related pathologies. However, the exact mechanism of SL in NASH has not been fully elucidated. We aimed to explore the therapeutic effect and potential mechanism of SL in NASH mice.

**Methods:**

We fed mice the methionine-choline deficiency (MCD) diet to construct a model of NASH. The efficacy of SL in regulating the progression of NASH was evaluated by biochemical and histopathological analyses. The effects of SL on oxidative stress and inflammatory cytokines were examined. Transcriptomic analysis was performed to further explore the potential molecular mechanisms regulated by SL. Finally, the expression of key proteins was verified by Western blotting.

**Results:**

The pathological phenotype of NASH was successfully established in mice. SL intervention demonstrated significant therapeutic benefits in reducing hepatic lipid accumulation, alleviating liver fibrosis, and decreasing inflammatory responses. Additionally, SL intervention was shown to ameliorate ER stress by attenuating the unfolded protein response (UPR). Gene expression profiling coupled with pathway analysis revealed that SL exerted its effects through the modulation of critical signalling pathways, including the PPAR signalling axis. Additionally, SL inhibited NLRP3 inflammasome activation and reduced the expression of downstream inflammatory cytokines in the liver.

**Discussion:**

The findings indicate that SL mitigates NASH by activating the PPAR pathway and suppressing the ER stress-triggered UPR, thereby inhibiting NLRP3 inflammasome activation. These results highlight the therapeutic potential of SL for NASH management.

## Graphical Abstract

**Figure:**
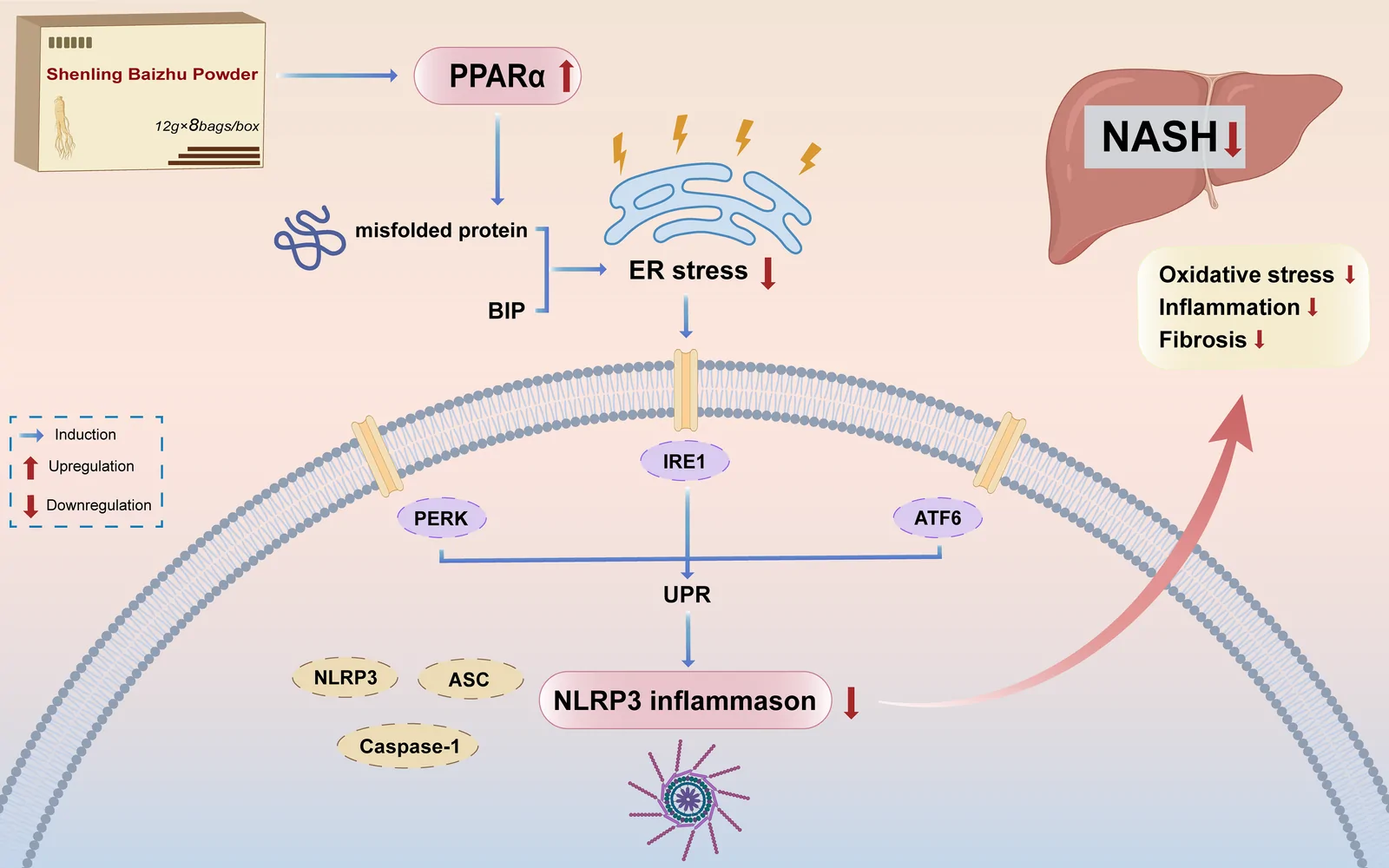

## 1 Introduction

The incidence and mortality of cirrhosis due to nonalcoholic fatty liver disease (NAFLD) are increasing in 16 regions of the world, making it the primary cause of cirrhosis-related mortality (1). According to epidemiological statistics, NAFLD extensively and profoundly affects approximately one-quarter of the global population (1–3). Nonalcoholic steatohepatitis (NASH) is characterized by hepatic steatosis, inflammation and fibrosis, which can develop into cirrhosis and HCC; thus, it has attracted much attention (4, 5). Recent data indicate a rising incidence of NASH (6), and while some drugs are believed to alleviate NASH (7, 8), very few have been approved for treating it (9), emphasizing the urgency of alternative treatment strategies.

The pathogenesis of NASH is complex and mostly involves inflammation, oxidative stress, lipid accumulation, insulin resistance and other factors (9, 10). Endoplasmic reticulum (ER) stress is a major component of oxidative stress and plays a crucial role in NASH (11–13).

Shenling Baizhu (SL) powder was first recorded in “Taiping Huimin Heji Jufang”, which was compiled during the Song Dynasty. This formula is composed exclusively of traditional Chinese medicines, such as *Panax ginseng* C.A. Mey., and *Poria cocos* (Schw.) Wolf, *Atractylodes macrocephala* Koidz., and *Dioscorea bulbifera* L. The entire formula can strengthen the spleen, benefit qi, infiltrate dampness and eliminate turbidity. It has been widely used to treat diseases for nearly a thousand years. SL exhibits significant efficacy in the management of gastrointestinal and liver lipid metabolism disorders (14–16), as it demonstrates pronounced ameliorative effects on inflammation, oxidative stress and lipid metabolism (17, 18). Studies have shown that SL significantly improves liver lipid deposition and oxidative stress damage, induced by a high-fat diet, in rat models of NAFLD (19). However, its potential to treat NASH and the underlying mechanisms remain unexplored. Given the pathophysiology of NASH, including excessive lipid accumulation, ER stress, and chronic inflammation, SL may play a beneficial role by targeting these processes.

NASH models were successfully induced in mice by administering a methionine-choline-deficient (MCD) diet, as described in our previous study (20). By combining biochemical analysis, histopathological analysis, transcriptomic analysis, and Western blotting, we aimed to elucidate the mechanisms by which SL improves liver lipid metabolism, reduces inflammation, and relieves ER stress. Our findings shed light on SL as a natural therapeutic agent that offers a novel pharmacological approach rooted in traditional medicine to address NASH and lipid metabolism disorders.

## 2 Materials and methods

### 2.1 Materials and chemicals

SL was prepared following traditional Chinese medicine formulations, and the detailed composition of the powder is presented in **Table 1**. LC–MS/MS was used for ingredient identification (**Supplementary Figure 1**) and the molecular weights of the top 10 components (**Table 1**). Polyene phosphatidylcholine (PPC) capsules were sourced from Sanofi Pharmaceuticals (Beijing, China). Biochemical reagents for measuring hepatic and serum markers were obtained from the Jiancheng Bioengineering Institute (Nanjing, China). ELISA kits used for cytokine detection were obtained from Multi Sciences Biotech (Hangzhou, China). The Antibodies against proteins, including NLRP3, ASC, Caspase-1, and β-actin, were obtained from Cell Signaling Technology (Massachusetts, USA). Anti-BIP, anti-IRE1, anti-PERK, and anti-ATF6 antibodies were provided by Bioss Antibodies (Beijing, China), and anti-PPARα antibodies were supplied by Abcam (Cambridge, USA). Staining kits for haematoxylin and eosin (HE), oil red O (ORO), and Masson’s trichrome were acquired from Servicebio Technology (Wuhan, China).

**Table 1:** The composition of Shenling Baizhu power.

Chinese name	Botanical name	Proportion
Ren Shen	*Panax ginseng* C.A. Mey.	5
Fu Ling	*Poria cocos* (Schw.) Wolf	5
Bai Zhu	*Atractylodes macrocephala* Koidz.	5
Bai Bian Dou	*Lablab purpureus subsp. purpureus*	4
Shan Yao	*Dioscorea bulbifera* L.	5
Gan Cao	*Glycyrrhiza glabra L. Preparata*	3
Lian Zi	*Nelumbinis plumula*	3
Sha Ren	*Amomum villosum* Lour.	2
Yi Yi Ren	*Coix lacryma-jobi* L.	3
Jie Geng	Platycodon *grandiforus*	2

### 2.2 Animal modelling and treatment

Trophic Animal Feed High-tech (Nantong, China) provided the MCD diet (60 kcal% fat, 0.1% methionine, and no choline supplementation) and the methionine-choline sufficient (MCS) diet (10 kcal% fat). Male C57BL/6J mice at 5 weeks of age were purchased from HFK Bioscience (Beijing, China). The control experimental temperature was 24 ± 2 °C, and the relative humidity was 55 ± 5%, with a 12-h light/dark cycle. After the final administration, mice were anesthetized with isoflurane in oxygen (3% at 1 L/min for induction and 1% at 1 L/min for maintenance). At the end of the experiment, euthanasia was performed using an overdose of isoflurane (5% in oxygen at 1 L/min) followed by exsanguination. All procedures complied with the ethical guidelines approved by the Institute of Laboratory Animal Resources, Jinan University (Guangzhou, China) (Licence No. IACUC-20220114-06).

The dosage of SL was selected as we previously described (21). After 10 days of adaptive feeding, all the mice were randomly distributed into 5 groups, with 10 mice in each group: the MCS group was fed an MCS diet for 3 weeks; the MCD group was fed an MCD diet for 3 weeks; and the MCD+SL-L group was fed 15 g/kg/d SL by gavage and fed an MCD diet for 3 weeks; the MCD+SL-H group was given 30 g/kg/d SL by gavage and fed an MCD diet for 3 weeks; and the MCD+PPC group was orally given 120 mg/kg/d PPC and fed an MCD diet for 3 weeks. Both the MCS group and the MCD group were administered the same amount of ionized water by gavage.

### 2.3 Surface microcirculation scan of the liver

After fasting for 12 hours prior to euthanasia, all the animals were anesthetized with pentobarbital (50 mg/kg). The mice were then exposed to normal temperature and natural light to measure liver blood flow. One frame per second is recorded for 60 seconds to generate the visual blood flow images.

### 2.4 Histological analysis

Liver tissues were coated with paraffin, sliced to 5 µm thickness, and stained with HE to evaluate overall histology. Lipid accumulation in tissues was assessed by ORO staining. The Masson method and α-SMA method were used to assess the degree of hepatic fibrosis. To assess the integrity and fine structure of ER, transmission electron microscopy (TEM) was used to compare ultrastructural changes among the different groups.

### 2.5 Biochemical detection

Blood samples were harvested, and the serum was isolated for subsequent analyses. The serum concentrations of ALT, AST, HDL-C and LDL-C were measured using corresponding kits. The supernatant of liver homogenate was collected after centrifugation, and the concentrations of TC, TG and NEFAs were determined by biochemical assay kits.

### 2.6 Oxidative stress index analysis

To assess liver oxidative stress, liver tissues were stained with dihydroethidium (DHE) according to the instructions of the kit. The reactive oxygen species (ROS) levels in the liver were subsequently evaluated by measuring the DHE fluorescence intensity under a fluorescence microscope. The homogenate was prepared by adding the sample homogenizer buffer of the kit to the homogenizer. The homogenate was then centrifuged at 12,000 rpm for 10 minutes to separate and collect the supernatant. The contents of SOD, MDA and GSH in the supernatant were subsequently determined following the kits’ instructions. Each experiment was repeated 6 times to ensure data reliability.

### 2.7 Cytokine analysis by ELISA

Total protein was extracted from the liver tissue homogenate, after which the supernatant was obtained by centrifugation. The levels of IL-1β, IL-6 and TNF-α in the liver were determined and quantitatively analysed following the instructions of the ELISA kits.

### 2.8 Liver transcriptomic analysis

Firstly, RNA integrity and concentration were assessed, and samples with RNA integrity number values >7.0 were then screened for clustering and quality control. A reference genome index was constructed and compared with the reference genome. The expression levels of the differentially expressed genes were analysed, and genes whose adjusted p value was <0.05 were defined as differentially expressed genes (DEGs). The overlapping differentially expressed genes identified from pairwise comparisons among the three groups were selected as the core gene set. Subsequently, functional annotation and pathway enrichment analyses were performed using GO-biological process (GO: BP) and the Kyoto Encyclopedia of Genes and Genomes (KEGG).

### 2.9 Western blot analysis

The total proteins from liver tissues were extracted with RIPA buffer, and the BCA method was used to determine the protein concentration. The target proteins were isolated and transferred to PVDF membranes using SDS–PAGE. After blocking, the membranes were incubated with primary antibodies at 4 °C overnight. After being washed with TBST, the membrane was incubated with secondary antibody for 1 h. The ECL detection system was used to visualize the protein bands, and ImageJ was used to quantify the protein expression levels.

### 2.10 Statistical analysis

GraphPad Prism 9.0 software was used for statistical analysis. Intergroup differences were tested by one-way ANOVA. The statistical results were expressed as the mean ± SD, and *p* < 0.05 was considered to indicate statistical significance.

## 3 Results

### 3.1 SL ameliorated lipid accumulation in MCD-induced NASH mice

**Figure 1:**
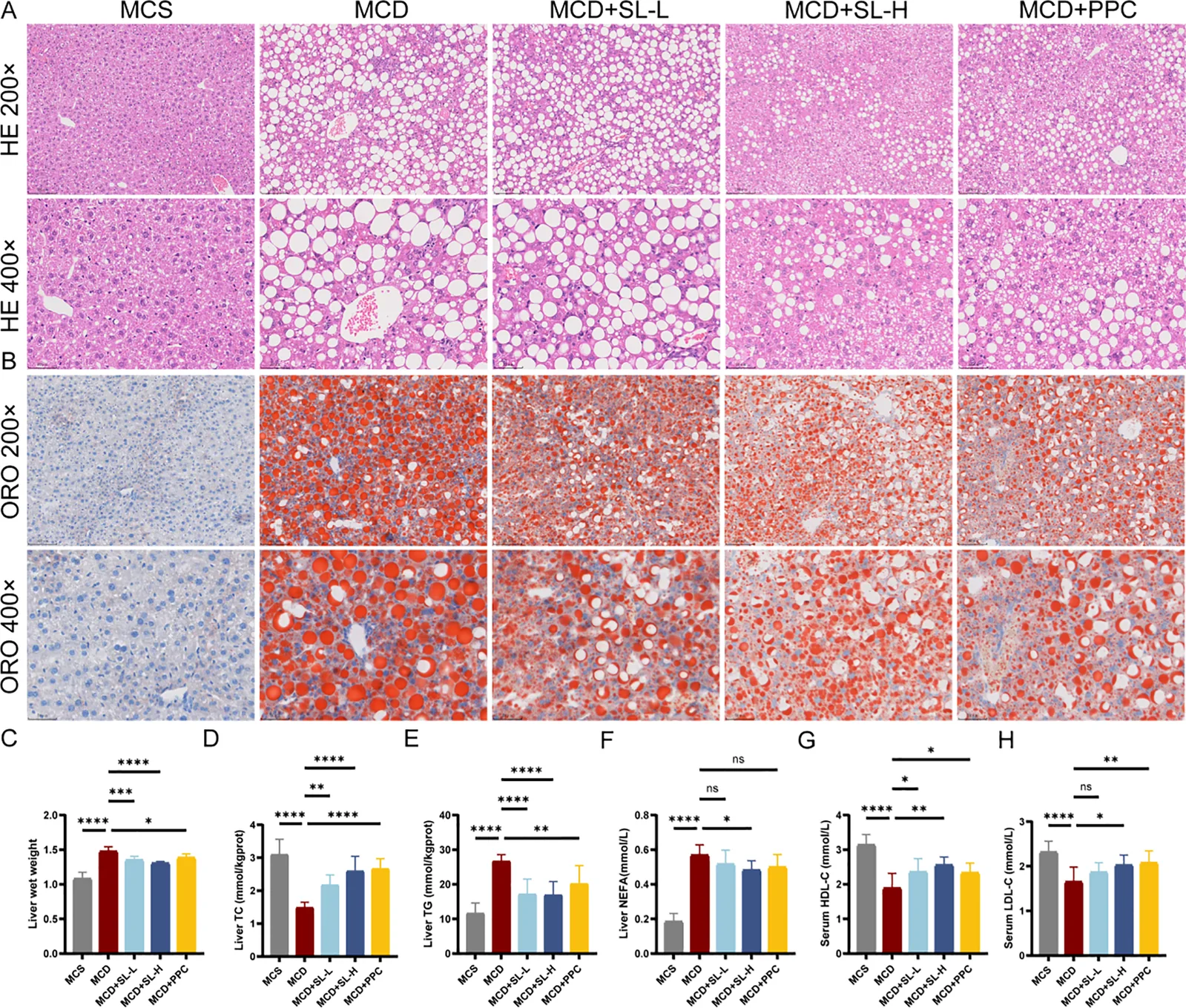
Liver pathological observation and biochemical parameters in mice. **(A)** Representative images of HE-stained sections (200 × and 400 ×). **(B)** Representative images of ORO staining (200 × and 400 ×). **(C)** Liver wet weight (n = 10 per group). **(D)** Liver TC levels (n = 10 per group). **(E)** Liver TG levels (n = 10 per group). **(F)** Liver NEFA levels (n = 10 per group). **(G)** Serum HDL-C levels (n = 10 per group). **(H)** Serum LDL-C levels (n = 10 per group). The data are presented as the mean ± SD. ^*^P < 0.05, ^**^P < 0.01, ^***^P < 0.001, *ns*, not significant.

Given that liver histopathological features are strong indicators for diagnosing NASH, we investigated the effects of SL on mice with NASH induced by 3 weeks of an MCD diet. After the mice were killed and fresh liver tissue was obtained, we assessed the extent of histopathological changes and lipid accumulation in the liver by HE and ORO staining (**Figures 1A, B**). Compared with those in the MCS group, the lipid droplets in the MCD group were irregularly arranged, and the size of the lipid droplets in hepatocytes was uneven and significantly increased, suggesting extensive degeneration of the liver tissue. After SL and PPC intervention, the above indices were compared with those in the MCD group. The above indices in the SL and PPC groups significantly improved. Notably, compared with the control group, the high-dose SL group exhibited significantly greater mitigation of lipid accumulation in the livers of NASH mice.

### 3.2 SL ameliorated biochemical parameters in mice with NASH

We also examined lipid metabolism and biochemical markers in mice. Compared with the MCS group, the MCD group exhibited significantly higher liver wet weight (**Figure 1C**), TG (**Figure 1E**) and NEFA (**Figure 1F**) levels; conversely, compared with the MCD group, the SL-treated group demonstrated a notable reduction in these parameters. Compared with the MCS group, the MCD group had significantly higher serum NEFA levels. SL-H treatment reduced NEFA levels, while SL-L showed a decreasing trend but without statistical significance versus the MCD group. The concentrations of TC (**Figure 1D**), HDL-C (**Figure 1G**) and LDL-C (**Figure 1H**) were highest in the MCS group and lowest in the MCD group. Upon treatment with SL or PPC, these lipid parameters were elevated compared to the MCD group, with the MCD+SL-H group demonstrating superior efficacy compared to the MCD+ SL-L group. These findings suggest that SL improves lipid metabolism in a dose-dependent manner, with higher doses yielding greater therapeutic benefits. These data suggest that SL and PPC restore normal liver lipid metabolism and effectively reduce liver damage in a dose-dependent manner.

### 3.3 SL reduced liver fibrosis in mice with NASH

**Figure 2:**
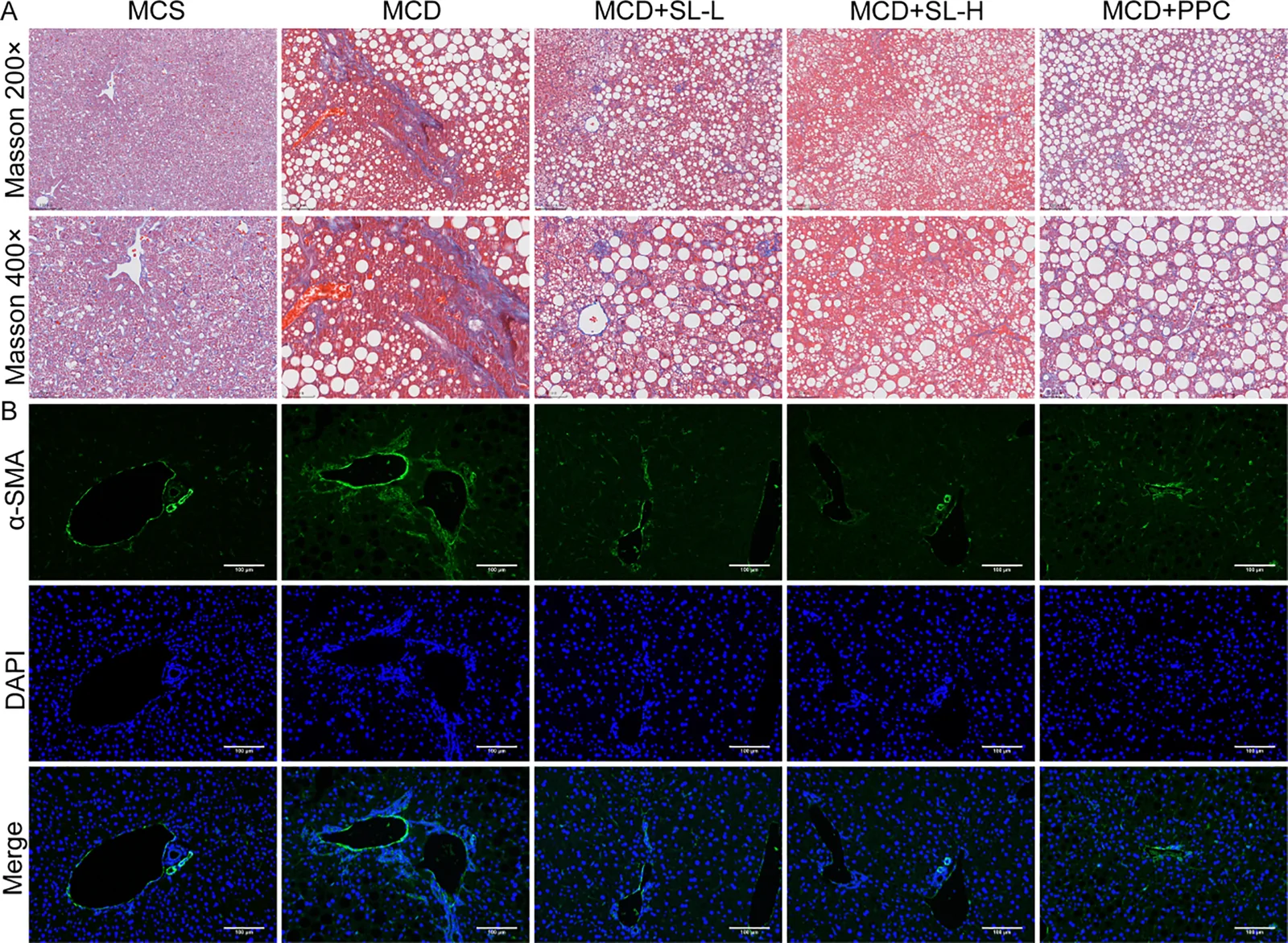
Effects of different interventions on liver fibrosis in NASH mice. **(A)** Representative Masson’s trichrome staining (200 × and 400 ×). **(B)** Immunofluorescence staining for α-SMA (green), a marker of activated hepatic stellate cells, counterstained with DAPI (blue) to indicate nuclei. Merged images highlight the distribution of α-SMA-positive cells. Scale bar, 100 μm.

Masson tricolour staining revealed significant collagen deposition in the MCD group. PPC and SL interventions significantly reduced fibrotic areas, with a more significant improvement in the MCD+SL-H group (**Figure 2A**). Compared with that in the MCD group, the expression of collagen fibre α-SMA was decreased in both the SL and PPC groups, especially in the SL-H group, indicating that SL had a stronger anti-fibrotic effect (**Figure 2B**).

### 3.4 SL reversed liver surface microcirculation disturbance in mice with NASH

**Figure 3:**
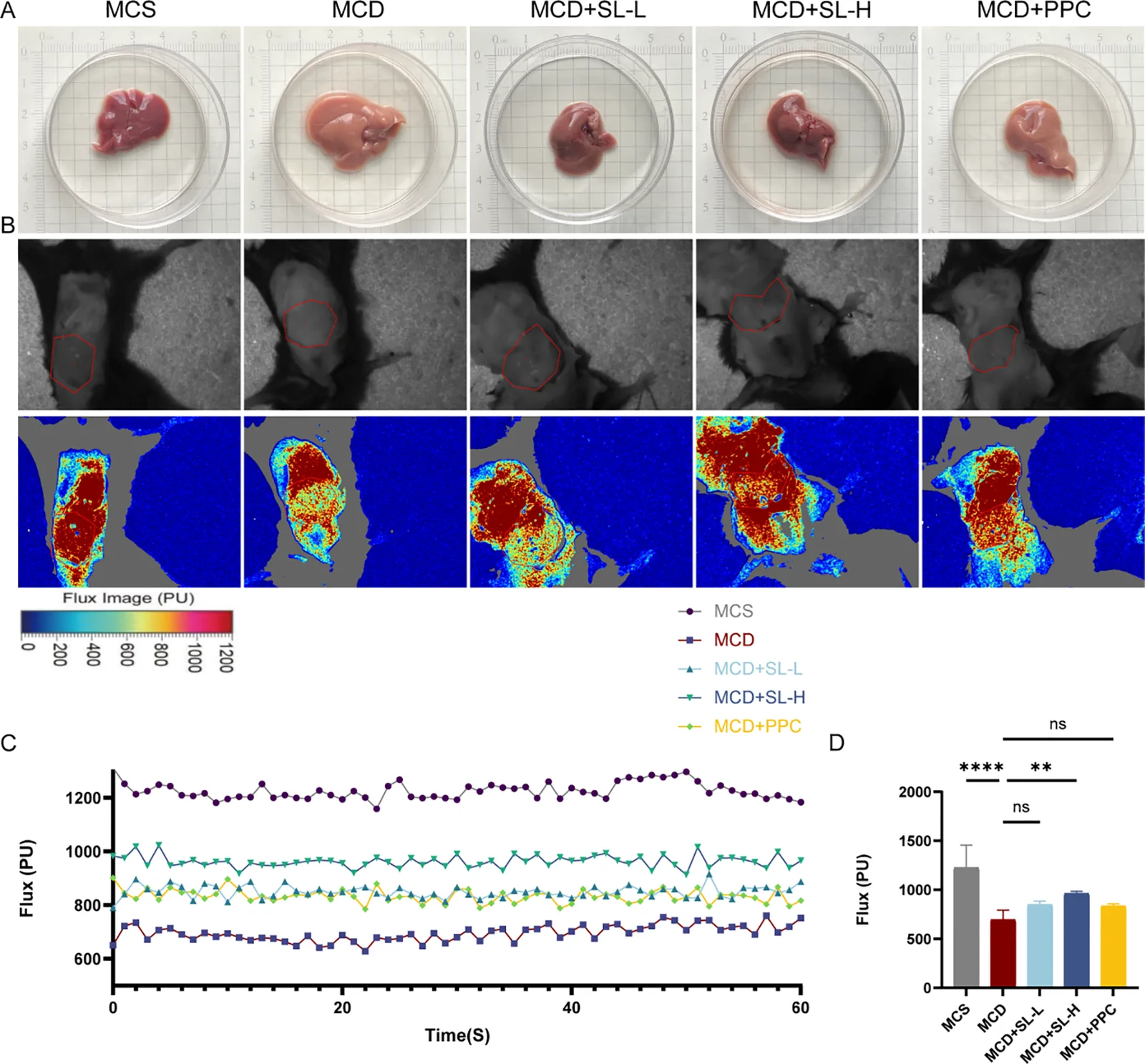
Liver surface blood flow scan. **(A)** Visual observation of the livers. **(B)** Scanning images of liver surface microcirculatory blood flow. The flux images (bottom row) represent blood PUs across the liver surface. The red and blue areas represent high- and low-perfusion areas, respectively. **(C)** The curves of liver microcirculatory blood flow in each experimental group were recorded, and the measurement time was 60 seconds. **(D)** Quantitative analysis of the average liver blood flow PU in each group (n = 10 per group). PU: perfusion units. The data are presented as the mean ± SD. ^*^P < 0.05, ^**^P < 0.01, ^***^P < 0.001, *ns*, not significant.

Mice in the MCD group had significantly enlarged livers with rough, greasy surfaces and blunt edges. These changes were notably reversed in both the low- and high-dose SL groups and in the PPC group (**Figure 3A**). Liver surface microcirculatory blood flow analysis revealed that blood flow was significantly reduced in the MCD group compared with the MCS group. The improvement was most significant in the MCD+SL-H group, indicating that the restoration of liver perfusion by SL or PPC was positively correlated with dose (**Figure 3B**). The results of the quantitative analysis also supported the above results, with those of the MCD+SL-H group more closely matching the levels of the MCS group (**Figures 3C, D**), suggesting that SL can reverse liver microcirculatory blood flow in mice with NASH, thereby inhibiting liver lipid accumulation.

### 3.5 RNA-seq analysis demonstrated that SL modulates NASH mice via the PPAR signaling pathway

**Figure 4:**
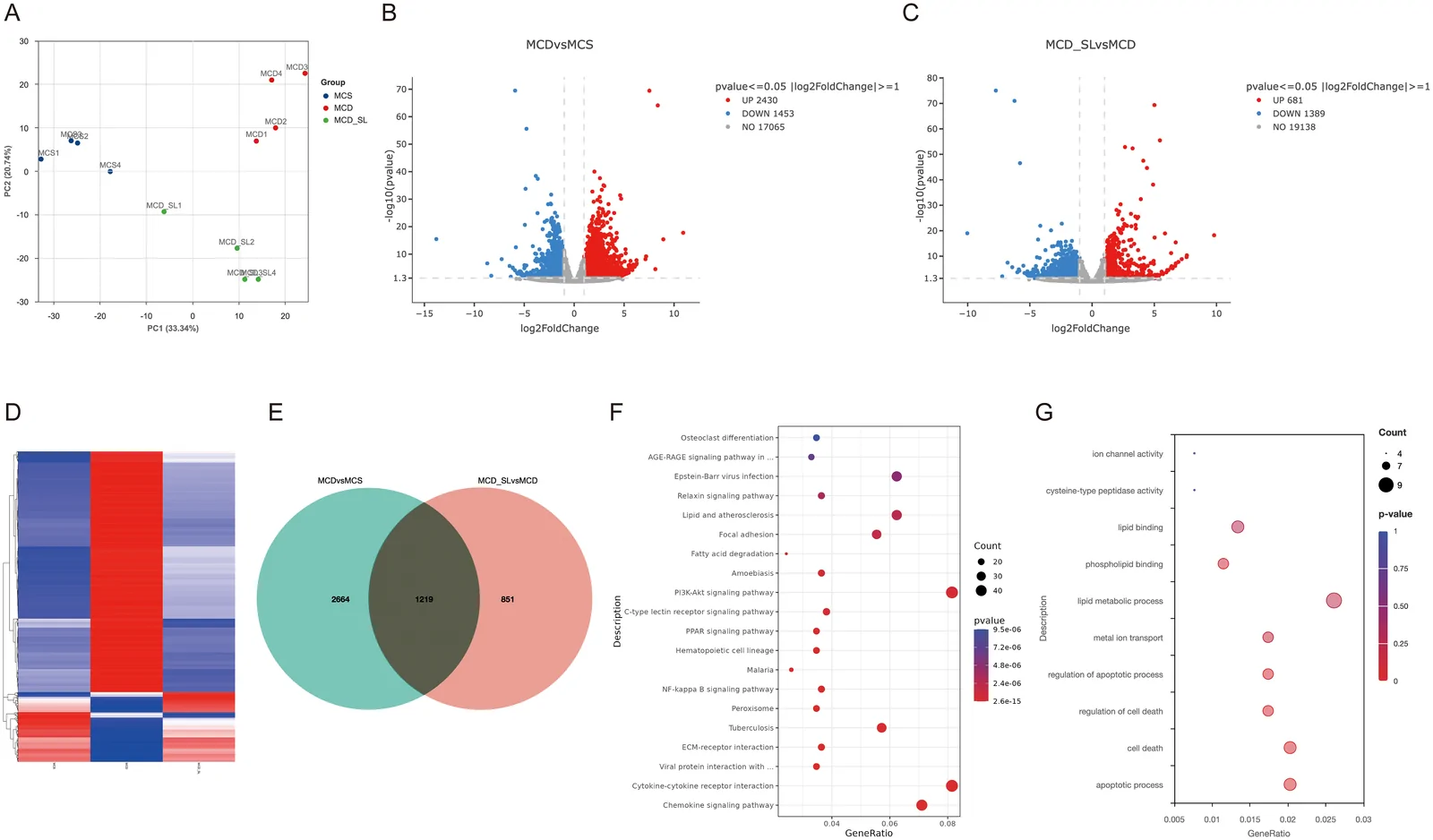
SL regulates the liver transcriptome of NASH mice. **(A)** Hepatic transcriptome PCA. **(B)** Volcano map of DEGs between the MCD group and the MCS group. **(C)** Volcano map of DEGs between the MCD_SL group and the MCD group. **(D)** Heatmap of differentially expressed genes. **(E)** Venn diagram of DEGs. **(F, G)** Based on the DEGs, KEGG and GO analyses were carried out, and the results are intuitively shown as bubble maps.

Next, transcriptomic analysis was performed on mice in the MCD, MCS and MCD+SL-H groups (hereafter referred to as the MCD_SL group). Principal component analysis (PCA) and heatmap analysis revealed significant differences between the MCD group, the MCS group, and the MCD_SL group, indicating that the MCD diet-induced NASH mice were successfully established (**Figures 4A–D**). To further elucidate the underlying mechanism by which SL acts on NASH mice, we performed RNA sequencing (RNA-seq). Among the MCS, MCD, and MCD_SL groups, 1219 genes were co-differentially expressed (**Figure 4E**). The KEGG enrichment pathway analysis performed specifically on these 1,219 overlapping DEGs revealed significant enrichment of multiple pathways, including the PPAR signaling pathway, the NF-κB signaling pathway and the PI3K-Akt signaling pathway. GO-biological process (GO: BP) analysis revealed significant enrichment of differentially expressed genes (DEGs) related to lipid metabolism and lipid binding processes (**Figures 4F, G**). In conclusion, SL may play a role in protecting the ER in NASH mice by regulating these key biological processes and signaling pathways.

### 3.6 SL mitigated oxidative stress and restored ER integrity in the livers of mice with NASH

**Figure 5:**
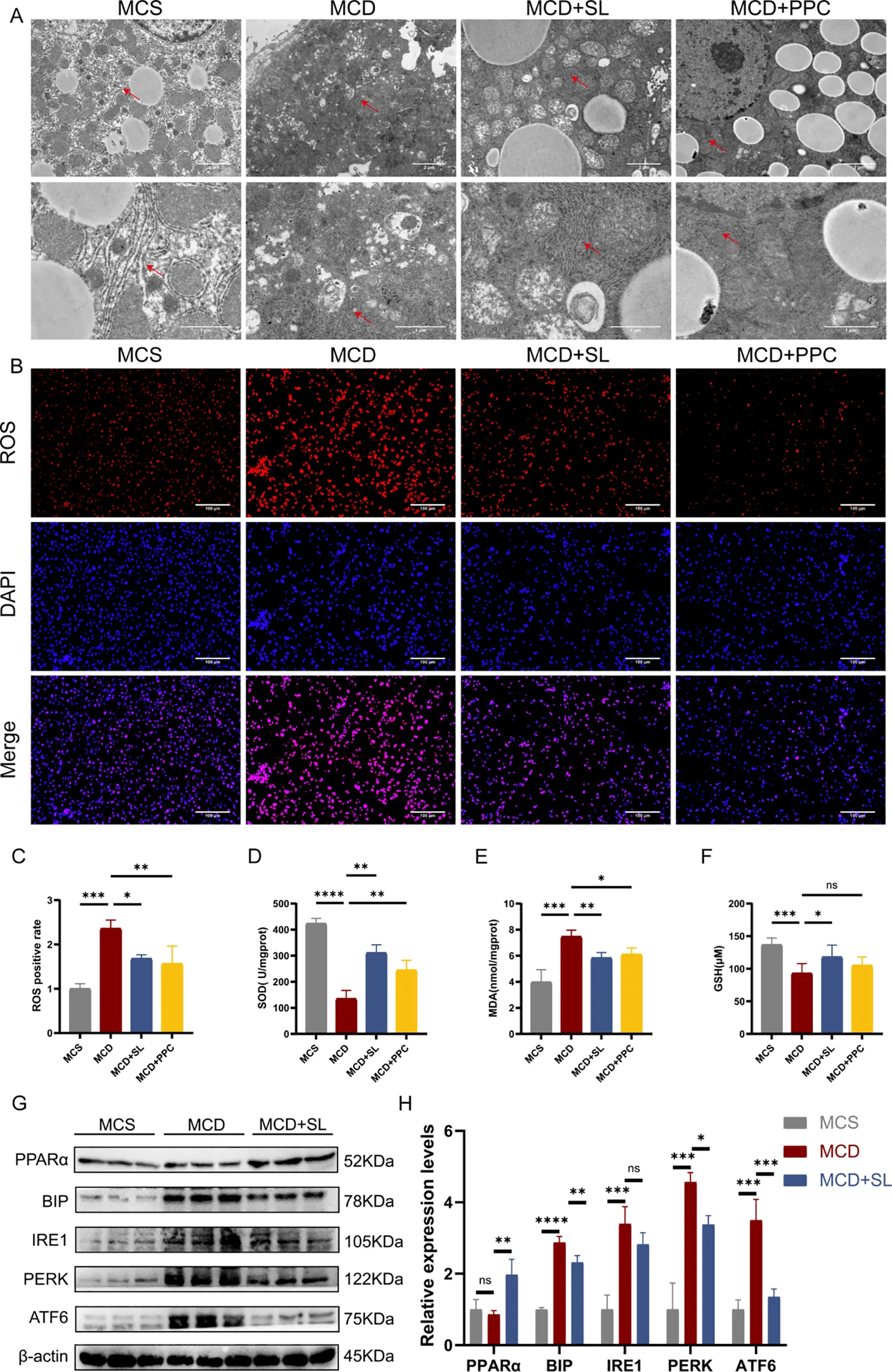
SL improved oxidative stress levels and ER ultrastructure in the livers of mice with NASH. **(A)** TEM images of liver tissue. Red arrows represent the ER (9700x and 23000x). **(B)** DHE staining of reactive ROS levels (scale bar, 50 μm). **(C–F)** Relative expression levels of ROS, SOD, MDA and GSH (n = 10 per group). **(G, H)** The protein expression levels of PPARα, BIP, IRE1, PERK and ATF6 were detected by Western blotting (n=3). The data are presented as the mean ± SD. ^*^P < 0.05, ^**^P < 0.01, ^***^P < 0.001, *ns*, not significant.

Disruption of endoplasmic reticulum homeostasis leads to ER stress, which is a well-established driver of NASH pathogenesis. Previous studies have shown that PPAR activation alleviates ER stress and mitigates NASH progression by reducing hepatic steatosis, fibrosis, and inflammation (22–24). Given that our RNA-seq analysis identified the PPAR signaling pathway as the predominant enriched pathway following SL intervention, we further evaluated the effect of SL on ER function. TEM revealed severe swelling of the ER, destruction of its structure ERand accumulation of large lipid droplets, in the MCD group. SL intervention, especially after high-dose SL treatment, significantly reduced lipid droplet deposition, alleviated ER swelling and restored its structural integrity (**Figure 5A**). We subsequently performed DHE staining and the determination of related oxidative stress indices, which revealed that the level of oxidative stress in the liver was more remarkable in the MCD group than in the control group, whereas SL and PPC markedly increased the level of oxidative stress in the livers of mice with NASH (**Figures 5B, C**). PPARα levels were significantly lower in the MCD group than in the control group, whereas the changes in BIP, IRE1, PERK, and ATF6 levels were reversed. However, these indicators were reversed in the MCD+SL group (**Figures 5D, F**). On the basis of these findings, we can further speculate that the remission of NASH from SL may negatively regulate the ER-associated UPR through the PPARα axis.

### 3.7 SL alleviated liver inflammation via inhibition of the NLRP3 inflammasome in mice with NASH

**Figure 6:**
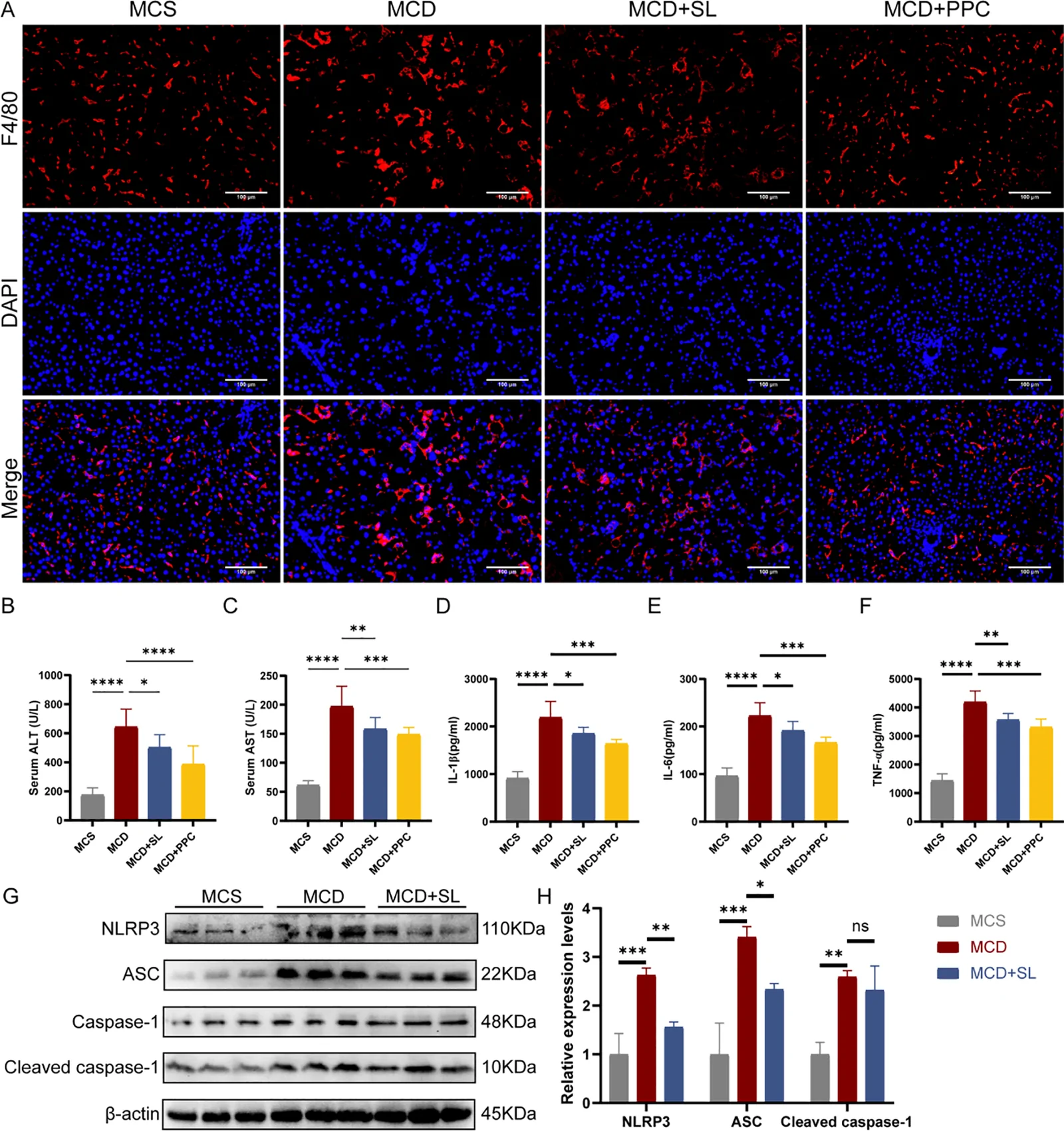
SL regulated the levels of inflammatory cytokines and activation of the NLRP3 inflammasome pathway in mice with NASH. **(A)** Immunofluorescence staining for the macrophage marker F4/80 (red) with nuclei counterstained using DAPI (blue), demonstrating macrophage infiltration in liver tissues (scale bar, 50 μm). **(B, C)** Serum ALT and AST levels (n = 10 per group). **(D–F)** Cytokine levels of IL-1β, IL-6, and TNF-α in liver tissues as measured by ELISA (n = 10 per group). **(G, H)** The protein expression levels of NLRP3, ASC, caspase-1 and cleaved caspase-1 were detected by Western blotting (n=3). The data are presented as the mean ± SD. ^*^P < 0.05, ^**^P < 0.01, ^***^P < 0.001, *ns*, not significant.

Immunofluorescence staining for F4/80 revealed obvious macrophage infiltration in the liver tissue of the MCD group, indicating an enhanced inflammatory response. Inflammatory infiltration was significantly reduced after SL treatment (**Figure 6A**). Liver function and inflammatory cytokine levels were markedly increased in the MCD group, but these markers were reversed by SL intervention (**Figures 6B–F**). Next, Western blotting was used to detect the expression of proteins related to the NLRP3 inflammasome. Compared with those in the MCS group, the expression levels of NLRP3, ASC, caspase-1 and cleaved caspase-1 in the MCD group were enhanced, indicating NLRP3 inflammasome activation. However, the expression of these indicators was notably inhibited by SL intervention. In summary, SL may exert an anti-inflammatory effect on NASH mice, possibly by inhibiting the activation of the NLRP3 inflammasome (**Figures 6G, H**).

## 4 Discussion

NASH is a growing global epidemic that progresses strongly to liver fibrosis and liver tumours. To date, very few drugs are approved for treating NASH (11). Hence, there is an urgent need for further development of novel pharmacological agents for treating NASH. Emerging evidence shows that traditional Chinese medicine has broad application prospects in the management of NASH, with its mechanisms of action involving multiple targets (25). The aim of this study was to explore the molecular mechanism of SL in the treatment of NASH by combining transcriptomics. Our study demonstrated that SL can improve NASH by reducing the activation of the ER stress-triggered UPR and NLRP3 inflammasome through the activation of PPARα.

Previous studies have confirmed that SL is effective against NAFLD by regulating the gut microbiota-liver axis and lipid droplet dynamics, or by inducing autophagy through specific compounds (14, 19, 21, 26, 27). Animal models induced by an MCD diet mimic the histological features of NASH because they readily progress from steatosis to steatohepatitis and even to fibrosis (28). In this study, mice were fed an MCD diet for 3 weeks to establish a NASH mouse model that successfully reproduced the characteristics of human NASH, including liver inflammation and metabolic disorders. Polyene phosphatidylcholine capsule (PPC), a compound containing large amounts of phospholipids from soybeans, has been reported to exert favourable anti-fatty liver and anti-fibrotic effects by alleviating liver injury and metabolic inflammation (29, 30). We followed the previous research experience and PPC was used as a positive control drug to evaluate the efficacy of SL in the NASH mouse model. Different doses of SL were used in the NASH mouse model, and the optimal intragastric dose of SL for the treatment of NASH was determined to be 30 g/kg (31, 32). The results of HE staining, ORO staining and biochemical indices revealed that SL could effectively increase liver lipid accumulation in NASH mice and reduce fat vacuole occurrence and ballooning. Masson staining and α-SMA immunofluorescence staining confirmed that SL effectively improved liver fibrosis in mice with NASH. Moreover, SL significantly improved liver microcirculation in mice with NASH. Overall, these data demonstrated that SL could alleviate the characteristic manifestations of NASH in mice.

As the metabolic centre of the human body, the liver plays an important role in maintaining metabolic homeostasis (33). We subsequently conducted liver transcriptomic analysis to further investigate the molecular mechanisms underlying the effects of SL against NASH in mice. The results indicated that the pathways through which SL was enriched in NASH mice included PPAR signaling pathway, NF-κB signaling pathway, PI3K-Akt signaling pathway, etc. Studies have shown that peroxisome proliferator-activated receptors (PPARs) play a key regulatory role in metabolism and inflammation (34). Abnormal expression of PPARs may lead to hepatic steatosis, NASH, fibrosis and HCC (35). The PPAR family includes three isoforms, namely, PPARα, PPARβ/δ and PPARγ (36). Among them, PPARα is expressed primarily in liver tissue and plays a central role in lipid metabolism (37, 38). Accumulating evidence has demonstrated that PPARα activation improves liver steatosis, inflammation, and fibrosis, serving as a potential therapeutic approach for treating NASH (39). As expected, our data revealed that SL intervention increased PPARα expression in the livers of NASH mice, indicating that SL may exert beneficial effects through PPAR activation. Notably, it is widely believed that PPARα serves as a crucial regulator for modulating lipid peroxidation. The ER (ER) is present inside cells and promotes the proper folding of thread-like peptides and proteins (40). The inability of the ER to form the correct protein 3D structure causes misfolded or unfolded proteins to accumulate within the ER, perturbs ER homeostasis, and causes ER stress (11, 41). Chronic ER stress has been shown to drive NAFLD progression (42, 43). The unfolded protein response (UPR) can trigger an inflammatory cascade, inflammasome activation, and hepatocyte apoptosis if ER stress is unresolved (44). Therefore, we next observed the ER structure in the livers of NASH mice. With SL intervention, most of the swollen ER was recovered, and lipid droplet aggregation was significantly reduced. Furthermore, the degree of oxidative stress has also been alleviated accordingly. Collectively, these findings suggest that SL can effectively mitigate oxidative stress and ER stress in the livers of NASH mice.

More recently, it has been reported that activating PPARα reduces ER stress-mediated UPR, thereby alleviating NASH progression (24). Three membrane-spanning UPR sensors, IRE1, PERK and ATF6, are responsible for sensing unfolded proteins within the ER lumen and relaying signalling cascades from the ER to the cytoplasm (45). The UPR mitigates ER stress by decreasing protein synthesis and upregulating the production of ER folding enzymes (46). Immunoglobulin binding protein (BIP) is a critical ER protein that is highly expressed in the ER. Under physiological conditions, BIP binds to and inhibits the transcriptional activity of all three UPR sensors (47). In response to ER stress, misfolded proteins interact with BIP, initiating its dissociation from the three UPR sensors. This dissociation activates the sensors and triggers their downstream signalling pathways, which are crucially involved in either restoring ER function or activating apoptotic pathways (48). To determine whether SL can trigger the UPR to improve NASH, the key proteins regulating the UPR were detected by Western blotting. As expected, we found that SL can upregulate PPARα and downregulate the expression levels of BIP, IRE1, PERK and ATF6, indicating that SL may exert an anti-NASH effect by activating PPARα and negatively regulating ER stress.

ER stress may induce the activation of the NLRP3 inflammasome in hepatocytes, leading to liver injury (49, 50). The NLRP3 inflammasome consists of NLRP3, ASC, and Caspase-1 proteins (51) and plays a significant role in the progression of NASH (52, 53). The NLRP3 inflammasome can mediate pyroptosis, inflammation and fibrosis in hepatocytes to affect the progression of NASH (54, 55). Therefore, the NLRP3 inflammasome is dependent on ER stress, and inhibiting its activation and the subsequent inflammatory cascade may be a potential strategy to treat NASH. In agreement with the findings of other studies (56, 57), our data revealed that the NLRP3 pathway was activated in the livers of NASH mice. On the other hand, the liver levels of IL-1β, IL-6 and TNF-α and the expression of NLRP3 family proteins in NASH mice were significantly inhibited by SL intervention. Taken together, these findings suggest that SL may inhibit NLRP3 activation by suppressing ER stress, thereby alleviating liver inflammation in NASH mice.

The present study has several limitations. Transcriptomic analysis revealed enrichment of the PPARα pathway following SL intervention, suggesting its potential involvement in the anti-NASH effects of SL. However, current data only show a correlation, and causality remains to be established using PPARα agonists/antagonists or gene silencing. Although SL modulated NLRP3 inflammasome-related pathways, direct evidence of ASC speck formation or Caspase-1 activity, the morphological gold standard for pyroptosome assembly and direct functional evidence of pyroptosis activation, respectively, is lacking. Thus, whether SL exerts anti-NASH effects via PPARα activation or pyroptosis inhibition requires further validation. Future studies should employ pharmacological and genetic interventions to clarify the role of PPARα.

## 5 Conclusions

In conclusion, this study reveals the significant potential of SL in alleviating NASH. These findings indicate that the mechanism of action of SL involves activating the PPARα pathway, negatively regulating ER stress-induced UPR, and inhibiting NLPR3 inflammasome activation. These actions reduced liver lipid accumulation, inflammation, and fibrosis, demonstrating that SL effectively modulates the progression of MCD diet-induced NASH.

## Data Availability

The datasets presented in this study can be found in online repositories. The names of the repository/repositories and accession number(s) can be found below: PRJNA1482749 (Bioproject, NCBI).

## Glossary

MCS: methionine-choline sufficient
MCD: methionine-choline deficiency
SL: Shenling Baizhu Powder
L: low
H: high
PPC: polyene phosphatidylcholine capsule
HE: hematoxylin and eosin
ORO: oil red O
TC: total cholesterol
TG: triglycerides
NEEA: non-esterified fatty acids
HDL-C: high-density lipoprotein cholesterol
LDL-C: low-density lipoprotein cholesterol. PCA, principal component analysis
DEGs: differentially expressed genes
KEGG: Kyoto encyclopedia of genes and genomes
RNA-seq: RNA sequencing
GO: BP, GO-biological process
PPAR: peroxisome proliferator-activated receptor
NF-κB: nuclear factor kappa-B
PI3K-Akt: phosphatidylinositol 3-kinase (PI3K)-Akt
TEM: transmission electron microscopy
DHE: dihydroethidium
ROS: reactive oxygen species
SOD: superoxide dismutase
MDA: malondialdehyde
GSH: glutathione
BIP: immunoglobulin binding protein
IRE1: inositol requiring protein-1
PERK: PKR-like ER kinase
ATF6: activating transcription factor-6. α-SMA, α-smooth muscle actin
DAPI: 4′,6-diamidino-2-phenylindole. ALT, alanine aminotransferase
AST: aspartate aminotransferase
IL-1β: interleukin-1β
IL-6: interleukin-6
TNF-α: tumor necrosis factor-α
NLRP3: NOD-like receptor thermal protein domain associated protein 3
ASC: apoptosis-associated speck-like protein containing a CARD

## References

[1] Paik JM Golabi P Younossi Y Mishra A Younossi ZM . Changes in the global burden of chronic liver diseases from 2012 to 2017: The growing impact of NAFLD. Hepatology. (2020) 72:1605–16. doi: 10.1002/hep.31173 32043613

[2] Younossi Z Anstee QM Marietti M Hardy T Henry L Eslam M . Global burden of NAFLD and NASH: trends, predictions, risk factors and prevention. Nat Rev Gastroenterol Hepatol. (2018) 15:11–20. doi: 10.1038/nrgastro.2017.109 28930295

[3] Younossi ZM Koenig AB Abdelatif D Fazel Y Henry L Wymer M . Global epidemiology of nonalcoholic fatty liver disease-meta-analytic assessment of prevalence, incidence, and outcomes. Hepatology. (2016) 64:73–84. doi: 10.1002/hep.28431 26707365

[4] Ioannou GN . The role of cholesterol in the pathogenesis of NASH. Trends Endocrinol Metab. (2016) 27:84–95. doi: 10.1016/j.tem.2015.11.008 26703097

[5] Sheka AC Adeyi O Thompson J Hameed B Crawford PA Ikramuddin S . Nonalcoholic steatohepatitis: A review. Jama. (2020) 323:1175–83. doi: 10.1001/jama.2020.2298 32207804

[6] Kanwal F Shubrook JH Younossi Z Natarajan Y Bugianesi E Rinella ME . Preparing for the NASH epidemic: A call to action. Gastroenterology. (2021) 161:1030–1042.e8. doi: 10.1053/j.gastro.2021.04.074 34416976

[7] Guo X Yin X Liu Z Wang J . Non-alcoholic fatty liver disease (NAFLD) pathogenesis and natural products for prevention and treatment. Int J Mol Sci. (2022) 23:15489. doi: 10.3390/ijms232415489 36555127 PMC9779435

[8] Wei S Wang L Evans PC Xu S . NAFLD and NASH: etiology, targets and emerging therapies. Drug Discov Today. (2024) 29:103910. doi: 10.1016/j.drudis.2024.103910 38301798

[9] Harrison SA Allen AM Dubourg J Noureddin M Alkhouri N . Challenges and opportunities in NASH drug development. Nat Med. (2023) 29:562–73. doi: 10.1038/s41591-023-02242-6 36894650

[10] Friedman SL Neuschwander-Tetri BA Rinella M Sanyal AJ . Mechanisms of NAFLD development and therapeutic strategies. Nat Med. (2018) 24:908–22. doi: 10.1038/s41591-018-0104-9 29967350 PMC6553468

[11] Ajoolabady A Kaplowitz N Lebeaupin C Kroemer G Kaufman RJ Malhi H . Endoplasmic reticulum stress in liver diseases. Hepatology. (2023) 77:619–39. doi: 10.1002/hep.32562 35524448 PMC9637239

[12] Dong J Chen L Ye F Tang J Liu B Lin J . Mic19 depletion impairs endoplasmic reticulum-mitochondrial contacts and mitochondrial lipid metabolism and triggers liver disease. Nat Commun. (2024) 15:168. doi: 10.1038/s41467-023-44057-6 38168065 PMC10762189

[13] Latif MU Schmidt GE Mercan S Rahman R Gibhardt CS Stejerean-Todoran I . NFATc1 signaling drives chronic ER stress responses to promote NAFLD progression. Gut. (2022) 71:2561–73. doi: 10.1136/gutjnl-2021-325013 35365570 PMC9664107

[14] Chen D Wang Y Yang J Ou W Lin G Zeng Z . Shenling Baizhu San ameliorates non-alcoholic fatty liver disease in mice by modulating gut microbiota and metabolites. Front Pharmacol. (2024) 15:1343755. doi: 10.3389/fphar.2024.1343755 38720776 PMC11076757

[15] Ma Q Ouyang Y Meng F Noolvi MN Avvaru SP More UA . A review of pharmacological and clinical studies on the application of Shenling Baizhu San in treatment of ulcerative colitis. J Ethnopharmacol. (2019) 244:112105. doi: 10.1016/j.jep.2019.112105 31344480

[16] Wang X Yang Q Zhou X Chen T Dou L Wang F . Shenling Baizhu powder inhibits RV-SA11-induced inflammation and rotavirus enteritis via TLR4/MyD88/NF-κB signaling pathway. Front Pharmacol. (2021) 12:642685. doi: 10.3389/fphar.2021.642685 33897431 PMC8062900

[17] Rao K Qin S Yang Y Zhan K Wu H Zheng H . Shenling Baizhu powder alleviates TNBS-induced colitis in rats by improving intestinal epithelial permeability and inhibiting inflammation through the TLR5/MyD88/NF-κB pathway. Front Pharmacol. (2022) 13:883918. doi: 10.3389/fphar.2022.883918 35571126 PMC9096158

[18] Yao Z Guo J Du B Hong L Zhu Y Feng X . Effects of Shenling Baizhu powder on intestinal microflora metabolites and liver mitochondrial energy metabolism in nonalcoholic fatty liver mice. Front Microbiol. (2023) 14:1147067. doi: 10.3389/fmicb.2023.1147067 37538846 PMC10394096

[19] Pan MX Zheng CY Deng YJ Tang KR Nie H Xie JQ . Hepatic protective effects of Shenling Baizhu powder, a herbal compound, against inflammatory damage via TLR4/NLRP3 signalling pathway in rats with nonalcoholic fatty liver disease. J Integr Med. (2021) 19:428–38. doi: 10.1016/j.joim.2021.07.004 34426178

[20] Liang Z Pi D Zhen J Yan H Zheng C Liang Chen J . The AMPK-mTOR pathway is inhibited by Chaihu Shugan powder, which relieves nonalcoholic steatohepatitis by suppressing autophagic ferroptosis. Mediators Inflammation. (2024) 2024:4777789. doi: 10.1155/2024/4777789 39502754 PMC11535263

[21] Pan M Deng Y Qiu Y Pi D Zheng C Liang Z . Shenling Baizhu powder alleviates non-alcoholic fatty liver disease by modulating autophagy and energy metabolism in high-fat diet-induced rats. Phytomedicine. (2024) 130:155712. doi: 10.1016/j.phymed.2024.155712 38763008

[22] Choi SW Oh H Park SY Cho W Abd El-Aty AM Baygutalp NK . Netrin-1 attenuates hepatic steatosis via UNC5b/PPARγ-mediated suppression of inflammation and ER stress. Life Sci. (2022) 311:121149. doi: 10.1016/j.lfs.2022.121149 36400204

[23] Xie X Liao Y Lin Z Luo H Wei G Huang N . Patchouli alcohol alleviates metabolic dysfunction-associated steatohepatitis via inhibiting mitochondria-associated endoplasmic reticulum membrane disruption-induced hepatic steatosis and inflammation in rats. Int Immunopharmacol. (2024) 138:112634. doi: 10.1016/j.intimp.2024.112634 38971107

[24] Pi D Liang Z Pan J Zhen J Zheng C Fan W . Tanshinone IIA inhibits the endoplasmic reticulum stress-induced unfolded protein response by activating the PPARα/FGF21 axis to ameliorate nonalcoholic steatohepatitis. Antioxidants (Basel). (2024) 13:1026. doi: 10.3390/antiox13091026 39334685 PMC11428933

[25] Chen M Xie Y Gong S Wang Y Yu H Zhou T . Traditional Chinese medicine in the treatment of nonalcoholic steatohepatitis. Pharmacol Res. (2021) 172:105849. doi: 10.1016/j.phrs.2021.105849 34450307

[26] Xia G Shen C Xiao Y Wang X Qiu L Lei S . Shenling Baizhu powder and betulin attenuate sepsis-induced intestinal injury by targeting GADD45B/TAOK1/p38 MAPK pathway. J Ethnopharmacol. (2025) 353:120282. doi: 10.1016/j.jep.2025.120282 40653040

[27] Cao L Wu Y Liu K-Y Qi N-X Zhang J Tie S-S . Cornus officinalis vinegar alters the gut microbiota, regulating lipid droplet changes in nonalcoholic fatty liver disease model mice. Food Med Homology. (2024) 1:9420002. doi: 10.26599/FMH.2024.9420002

[28] Ning M Lu D Teng B Liang D Ren PG . Comprehensive study of the murine MASH models' applicability by comparing human liver transcriptomes. Life Sci. (2025) 376:123723. doi: 10.1016/j.lfs.2025.123723 40404118

[29] Lu Y Feng T Zhao J Jiang P Xu D Zhou M . Polyene phosphatidylcholine ameliorates high fat diet-induced non-alcoholic fatty liver disease via remodeling metabolism and inflammation. Front Physiol. (2022) 13:810143. doi: 10.3389/fphys.2022.810143 35295576 PMC8918669

[30] Mak KM Shekhar AC . Soybean polyenylphosphatidylcholine (PPC) is beneficial in liver and extrahepatic tissue injury: An update in experimental research. Anat Rec (Hoboken). (2024) 307:2162–86. doi: 10.1002/ar.25333 37814787

[31] Liu Y Tan Y Huang J Wu C Fan X Stalin A . Revealing the mechanism of Huazhi Rougan granule in the treatment of nonalcoholic fatty liver through intestinal flora based on 16S rRNA, metagenomic sequencing and network pharmacology. Front Pharmacol. (2022) 13:875700. doi: 10.3389/fphar.2022.875700 35559233 PMC9086680

[32] Qi W Cao X Chen Y Chen H Zhang N Liu R . JiGuCao capsule formula alleviates metabolic fatty liver disease by regulating the gut-liver axis and lipid metabolism. Phytomedicine. (2025) 140:156559. doi: 10.1016/j.phymed.2025.156559 40064115

[33] Ronca V Gerussi A Collins P Parente A Oo YH Invernizzi P . The liver as a central "hub" of the immune system: pathophysiological implications. Physiol Rev. (2025) 105:493–539. doi: 10.1152/physrev.00004.2023 39297676

[34] Lange NF Graf V Caussy C Dufour JF . PPAR-targeted therapies in the treatment of non-alcoholic fatty liver disease in diabetic patients. Int J Mol Sci. (2022) 23:4305. doi: 10.3390/ijms23084305 35457120 PMC9028563

[35] Gross B Pawlak M Lefebvre P Staels B . PPARs in obesity-induced T2DM, dyslipidaemia and NAFLD. Nat Rev Endocrinol. (2017) 13:36–49. doi: 10.1038/nrendo.2016.135 27636730

[36] Christofides A Konstantinidou E Jani C Boussiotis VA . The role of peroxisome proliferator-activated receptors (PPAR) in immune responses. Metabolism. (2021) 114:154338. doi: 10.1016/j.metabol.2020.154338 32791172 PMC7736084

[37] Bougarne N Weyers B Desmet SJ Deckers J Ray DW Staels B . Molecular actions of PPARα in lipid metabolism and inflammation. Endocr Rev. (2018) 39:760–802. doi: 10.1210/er.2018-00064 30020428

[38] Tahri-Joutey M Andreoletti P Surapureddi S Nasser B Cherkaoui-Malki M Latruffe N . Mechanisms mediating the regulation of peroxisomal fatty acid beta-oxidation by PPARα. Int J Mol Sci. (2021) 22:8969. doi: 10.3390/ijms22168969 34445672 PMC8396561

[39] Pawlak M Lefebvre P Staels B . Molecular mechanism of PPARα action and its impact on lipid metabolism, inflammation and fibrosis in non-alcoholic fatty liver disease. J Hepatol. (2015) 62:720–33. doi: 10.1016/j.jhep.2014.10.039 25450203

[40] Celik C Lee SYT Yap WS Thibault G . Endoplasmic reticulum stress and lipids in health and diseases. Prog Lipid Res. (2023) 89:101198. doi: 10.1016/j.plipres.2022.101198 36379317

[41] Chen X Shi C He M Xiong S Xia X . Endoplasmic reticulum stress: molecular mechanism and therapeutic targets. Signal Transduct Target Ther. (2023) 8:352. doi: 10.1038/s41392-023-01570-w 37709773 PMC10502142

[42] Liu C Zhou B Meng M Zhao W Wang D Yuan Y . FOXA3 induction under endoplasmic reticulum stress contributes to non-alcoholic fatty liver disease. J Hepatol. (2021) 75:150–62. doi: 10.1016/j.jhep.2021.01.042 33548387

[43] Nasiri-Ansari N Nikolopoulou C Papoutsi K Kyrou I Mantzoros CS Kyriakopoulos G . Empagliflozin attenuates non-alcoholic fatty liver disease (NAFLD) in high fat diet fed ApoE((-/-)) mice by activating autophagy and reducing ER stress and apoptosis. Int J Mol Sci. (2021) 22:818. doi: 10.3390/ijms22020818 33467546 PMC7829901

[44] Lebeaupin C Vallée D Hazari Y Hetz C Chevet E Bailly-Maitre B . Endoplasmic reticulum stress signalling and the pathogenesis of non-alcoholic fatty liver disease. J Hepatol. (2018) 69:927–47. doi: 10.1016/j.jhep.2018.06.008 29940269

[45] Prasad V Greber UF . The endoplasmic reticulum unfolded protein response - homeostasis, cell death and evolution in virus infections. FEMS Microbiol Rev. (2021) 45:fuab016. doi: 10.1093/femsre/fuab016 33765123 PMC8498563

[46] Frakes AE Dillin A . The UPR(ER): Sensor and coordinator of organismal homeostasis. Mol Cell. (2017) 66:761–71. doi: 10.1016/j.molcel.2017.05.031 28622521

[47] Kopp MC Larburu N Durairaj V Adams CJ Ali MMU . UPR proteins IRE1 and PERK switch BiP from chaperone to ER stress sensor. Nat Struct Mol Biol. (2019) 26:1053–62. doi: 10.1038/s41594-019-0324-9 31695187 PMC6858872

[48] Xia SW Wang ZM Sun SM Su Y Li ZH Shao JJ . Endoplasmic reticulum stress and protein degradation in chronic liver disease. Pharmacol Res. (2020) 161:105218. doi: 10.1016/j.phrs.2020.105218 33007418

[49] Han CY Rho HS Kim A Kim TH Jang K Jun DW . FXR inhibits endoplasmic reticulum stress-induced NLRP3 inflammasome in hepatocytes and ameliorates liver injury. Cell Rep. (2018) 24:2985–99. doi: 10.1016/j.celrep.2018.07.068 30208322

[50] Han D Kim H Kim S Le QA Han SY Bae J . Sestrin2 protects against cholestatic liver injury by inhibiting endoplasmic reticulum stress and NLRP3 inflammasome-mediated pyroptosis. Exp Mol Med. (2022) 54:239–51. doi: 10.1038/s12276-022-00737-9 35260799 PMC8980001

[51] Fu J Wu H . Structural mechanisms of NLRP3 inflammasome assembly and activation. Annu Rev Immunol. (2023) 41:301–16. doi: 10.1146/annurev-immunol-081022-021207 36750315 PMC10159982

[52] de Carvalho Ribeiro M Szabo G . Role of the inflammasome in liver disease. Annu Rev Pathol. (2022) 17:345–65. doi: 10.1146/annurev-pathmechdis-032521-102529 34752711 PMC10501045

[53] Mangan MSJ Olhava EJ Roush WR Seidel HM Glick GD Latz E . Targeting the NLRP3 inflammasome in inflammatory diseases. Nat Rev Drug Discov. (2018) 17:688. doi: 10.1038/nrd.2018.149 30116046

[54] Mridha AR Wree A Robertson AAB Yeh MM Johnson CD Van Rooyen DM . NLRP3 inflammasome blockade reduces liver inflammation and fibrosis in experimental NASH in mice. J Hepatol. (2017) 66:1037–46. doi: 10.1016/j.jhep.2017.01.022 28167322 PMC6536116

[55] Wang Q Ou Y Hu G Wen C Yue S Chen C . Naringenin attenuates non-alcoholic fatty liver disease by down-regulating the NLRP3/NF-κB pathway in mice. Br J Pharmacol. (2020) 177:1806–21. doi: 10.1111/bph.14938 31758699 PMC7070172

[56] Deng YF Xu QQ Chen TQ Ming JX Wang YF Mao LN . Kinsenoside alleviates inflammation and fibrosis in experimental NASH mice by suppressing the NF-κB/NLRP3 signaling pathway. Phytomedicine. (2022) 104:154241. doi: 10.1016/j.phymed.2022.154241 35749827

[57] Yang W Liu L Wei Y Fang C Liu S Zhou F . Exercise suppresses NLRP3 inflammasome activation in mice with diet-induced NASH: a plausible role of adropin. Lab Invest. (2021) 101:369–80. doi: 10.1038/s41374-020-00508-y 33268842

