# Supplementary material for: Shenling Baizhu powder alleviates non-alcoholic steatohepatitis by reducing PPARα-mediated NLRP3 inflammasome activation
Source: Front Immunol. 2026 Aug 20;17:1797561. doi: 10.3389/fimmu.2026.1797561 (PMC13539331; doi:10.3389/fimmu.2026.1797561)
Supplement: Supplementary file 1 [file DataSheet1.docx]

**Supplementary FIGURE S1**

UPLC-MS/MS was used to identify the components of SL. **(A)** Total ions diagram of mixed quality control sample. **(B)** Multimodal map of MRM metabolite detection. **(C)** TIC overlap was detected by QC sample essence spectrum. N and P represent the negative and positive ion mode respectively.

**Supplementary Table S1** The top 10 molecular weight compounds in SL.

| **Compounds** | **Molecular weight (Da)** |
| --- | --- |
| Chikusetsusaponin Fk4 | 1210.6346 |
| Malonyl floralginsenoside Rb2 | 1194.6033 |
| Methyl-soyasapogenol B-Rha-GlcA-GlcA-Glc | 1132.5302 |
| Yunganogenin F-GlcA-GlcA-Glc-Rha | 1130.5145 |
| Ginsenoside Rb1 | 1108.6029 |
| Deapi-platycodin D | 1092.5353 |
| Medicagenic acid-3-O-glucuronide-28-O-xylosyl (1,4)-rhamnosyl (1,2)-arabinoside | 1088.504 |
| Notoginsenoside Lb | 1078.5924 |
| Ginsenoside Rb3 | 1078.5924 |
| Floralginsenoside N | 1078.5924 |
